# Adiponectin Receptor Agonist Ameliorates Synaptic Dysfunction in 3xTg Alzheimer's Disease Mouse Model by Activation of AMPK

**DOI:** 10.1111/cns.70616

**Published:** 2025-09-22

**Authors:** Jenna Bloemer, Priyanka D. Pinky, Vishnu Suppiramaniam, Miranda N. Reed

**Affiliations:** ^1^ Department of Pharmaceutical Sciences, College of Pharmacy Larkin University Miami Florida USA; ^2^ Department of Drug Discovery and Development Auburn University Auburn Alabama USA; ^3^ Center for Neuroscience Initiative Auburn University Auburn Alabama USA; ^4^ Department of Molecular and Cellular Biology, College of Science and Mathematics Kennesaw State University Kennesaw Georgia USA

**Keywords:** adiponectin, Alzheimer's disease, AMPK, synaptic plasticity

## Abstract

**Aim:**

The hormone adiponectin impacts various facets of brain function, including neurogenesis, energy homeostasis, and synaptic processes. The use of adiponectin or adiponectin receptor agonists may protect against Alzheimer's disease (AD) and reduce AD pathology. Here, we investigated the ability of the adiponectin receptor agonist, AdipoRon, to restore synaptic function in an AD mouse model and the underlying mechanism.

**Methods:**

Acute hippocampal slices from 3xTg‐AD mice and age‐matched controls were used to evaluate the ability of AdipoRon to rescue synaptic deficits in an AD model. Slices were incubated in AdipoRon or other pharmacological agents, followed by electrophysiological field recordings to evaluate synaptic function and plasticity. Signaling pathway alterations were evaluated by Western blot, with a focus on AMP‐activated protein kinase (AMPK) signaling.

**Results:**

Incubation of hippocampal slices with AdipoRon ameliorated long‐term potentiation (LTP) and basal synaptic transmission deficits in 3xTg‐AD mice. AdipoRon was unable to restore these parameters in the presence of the AMPK inhibitor, Compound C. AdipoRon altered presynaptic parameters by a mechanism that did not appear to be solely dependent on AMPK. AdipoRon slice incubation was associated with activation of AMPK, inhibition of GSK3β, and altered glutamatergic receptor subunit phosphorylation based on Western blot analysis.

**Conclusion:**

Activation of adiponectin receptors restores synaptic function in an AD model in part through AMPK signaling. These results warrant further investigation into adiponectin receptor agonists as a novel approach for AD prevention or treatment.

## Graphical abstract

The adiponectin receptor agonist AdipoRon improves synaptic function in the 3xTg AD mouse model based on electrophysiological recordings following hippocampal slice incubation. Enhancement of synaptic plasticity and basal synaptic transmission is blocked by an AMPK inhibitor. Improvement in synaptic function is associated with AMPK‐mediated inactivation of GSK3β and phosphorylation of the glutamatergic receptor subunit GluA1. Created in BioRender. Bloemer, J. (2025) https://BioRender.com/m44h123.

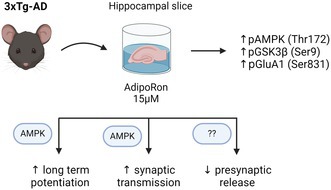

## 1 Introduction

Adiponectin is a hormone that influences brain processes, including hippocampal neurogenesis and brain energy homeostasis [1, 2]. There appears to be an association between altered adiponectin levels and Alzheimer's disease (AD). Some reports indicate that adiponectin levels in the cerebral spinal fluid (CSF) are reduced in AD [3, 4], and plasma adiponectin levels predict cognitive decline and cortical thinning in mild cognitive impairment with beta‐amyloid pathology [5]. Preclinical studies indicate a neuroprotective role of adiponectin receptor signaling against AD, as a reduction in adiponectin receptor expression in the brain leads to an AD‐like pathology [6], and adiponectin receptor agonists reduce neuropathology in animal models of AD [7, 8, 9]. Thus, there is interest in the use of adiponectin receptor agonists in the prevention or treatment of AD.

AD is characterized by numerous pathological alterations, including neuroinflammation, brain insulin resistance, synaptic dysfunction, and amyloid‐beta (Aβ)/tau‐mediated toxicity [10], and there is evidence that enhanced adiponectin signaling may reduce these pathologies [8, 11, 12]. Synaptic dysfunction is a key component of AD pathology, and impairment in long‐term potentiation (LTP) is a characteristic feature of animal models of AD [13]. Adiponectin receptor signaling influences synaptic processes [14], and treatment with adiponectin rescues synaptic plasticity and cognitive deficits in AD mouse models [15, 16], however, the specific mechanism for synaptic improvement is not fully elucidated. Adiponectin activates multiple signaling molecules involved in synaptic function, including 5′ AMP‐activated protein kinase (AMPK), peroxisome proliferator‐activated receptor (PPARα), and phosphatidylinositol 3‐kinase and protein kinase B (PI3k/Akt). Thus, the goal of the current study was to determine whether acute adiponectin receptor activation can reverse synaptic impairments in 3xTg‐AD mice and to examine if this effect is dependent upon AMPK, which is inhibited by Aβ oligomers [17] and a major downstream signaling molecule of AdipoR1. Utilizing hippocampal slices from 8 to 9‐month‐old 3xTg‐AD and control mice, we evaluated the ability of AdipoRon, an agonist at AdipoR1 and AdipoR2, to rescue synaptic deficits. We also determined whether inhibition of AMPK could prevent the effects of AdipoRon on synaptic processes. Our results indicate that acute restoration of adiponectin receptor signaling rescues deficits in synaptic plasticity and basal synaptic transmission through activation of AMPK.

## 2 Materials and Methods

### 2.1 Animals

3xTg‐AD and non‐transgenic control mice were obtained from The Jackson Laboratory and bred at an in‐house facility. Mice were group‐housed with free access to food and water in a temperature‐ and humidity‐controlled colony room with a 12:12 light/dark cycle. Female 3xTg‐AD and control mice were used for all experiments due to a lack of phenotypic traits in male transgenic mice (The Jackson Laboratory). For electrophysiology experiments, brain slices from 8 to 9‐month‐old mice were used. All procedures were carried out in accordance with NIH guidelines and approved by the Auburn University Animal Care and Use Committee.

### 2.2 Chemicals

AdipoRon (Cayman Chemical) was dissolved in dimethyl sulfoxide (DMSO) and stored as a stock solution, then diluted in artificial cerebral spinal fluid (ACSF) on the day of the experiment to a final concentration of 15 μM (Zhang et al. 2017). 5‐Aminoimidazole‐4‐carboxamide ribonucleotide (AICAR) (Cayman Chemical) was diluted in ACSF to a final concentration of 1 mM [18]. Compound C, also known as dysmorphin (Cayman Chemical), was dissolved in DMSO and stored as a stock solution, then diluted in ACSF on the day of the experiment to a final concentration of 10 μM [18]. All other chemicals, including those used to make ACSF, were obtained from Millipore Sigma, unless otherwise specified.

### 2.3 Immunoblotting

Hippocampal tissue was homogenized in lysis buffer (Radioimmunoprecipitation assay buffer, ThermoFischer Scientific) containing protease and phosphatase inhibitor cocktail (Halt Protease and Phosphatase Inhibitor Cocktail, ThermoFischer Scientific). Total protein was estimated by bicinchoninic acid (BCA) assay (Pierce BCA Protein Assay Kit, ThermoFischer Scientific) and stored at −20°C until use. Samples were mixed thoroughly with 4× Laemmli buffer, heated, and loaded into a handcast 10% SDS‐PAGE gel. Electrophoresis was performed using the Mini‐PROTEAN 3 system (Bio‐Rad). The proteins were transferred to PVDF membranes (Amersham Hybond P, GE Healthcare) and blocked with 5% bovine serum albumin (BSA) in Tris‐buffered saline, 0.1% v/v Tween 20 (TBST) for 2 h. Prior to antibody incubation, the blots were cut into thin strips based on the molecular weight of the probed protein (visualized by Precision Plus Protein All Blue Prestained Protein Standards—BioRad, Benicia, CA, USA) to probe for multiple proteins on the same blot and reduce the required incubation volume. Membranes were washed with TBST and incubated with primary antibody overnight at 4°C. Additional washing steps were performed, followed by incubation in horseradish peroxidase‐conjugated secondary antibody for 1 h at room temperature. Primary antibodies and secondary antibodies were diluted in 5% BSA and utilized at the concentrations indicated in Table 1. Immunoreactivity was visualized using enhanced chemiluminescence (Amersham ECL Select or Amersham ECL Prime, GE Healthcare) in a FluorChem Q imager system (ProteinSimple). Density of immunoreactivity for each band was measured using AlphaView software (ProteinSimple), and beta actin was used as a loading control.

**TABLE 1:** Summary of antibodies and working conditions used in the experiments.

Antibodies	Species	Source	Catalogue #	Dilution
Primary antibodies				
AMPK	Rabbit	Cell Signaling Technology	5832	1:1000
pAMPK (Thr 172)	Rabbit	Cell Signaling Technology	2531	1:500
GSKβ	Rabbit	Cell Signaling Technology	12,456	1:1000
pGSK3β (Ser 9)	Rabbit	Cell Signaling Technology	5558	1:1000
GluA1	Rabbit	Cell Signaling Technology	13,185	1:750
pGluA1 (Ser831)	Rabbit	Cell Signaling Technology	75,574	1:500
Beta Actin	Rabbit	Cell Signaling Technology	8457	1:2000
AdipoR1	Rabbit	Abcam	ab126611	1:200
AdipoR2	Mouse	Santa Cruz	sc‐514,045	1:200
Adiponectin	Rabbit	Abcam	ab62551	1:200
Secondary antibodies				
Anti‐mouse IgG	N/A	Santa Cruz	sc‐516,102	1:2000
Anti‐rabbit IgG	Goat	Cell Signaling Technology	7074	1:5000

### 2.4 Enzyme‐Linked Immunosorbent Assay (ELISA)

Whole blood samples were collected from 4‐, 8‐, and 12‐month‐old mice. All samples were collected at the same time of day to reduce the impact of diurnal variations on adiponectin levels. Samples were collected in tubes and left to clot at room temperature for 90 min. Next, samples were centrifuged at 2000 × g for 15 min at room temperature. The supernatant (serum) was collected immediately following centrifugation and stored at −80°C until use. Assay was performed following the standard instructions provided by the company (Cayman Chemical, Adiponectin (mouse) ELISA Kit).

### 2.5 Hippocampal Slice Preparation

Mice were euthanized with carbon dioxide, and 350 μm thick coronal slices were prepared using a Leica VT1200S Vibratome (Leica Microsystems, Wetzlar, Germany). Slices were incubated at room temperature in ACSF (124 mM NaCl, 2.5 mM KCl, 1.5 mM MgCl_2_, 2 mM CaCl_2_, 1.25 mM NaH_2_PO_4_, 25 mM NaHCO_3_, 25 mM dextrose, pH 7.4) saturated with 95% O_2_/5% CO_2_ until transfer to the recording chamber. For all electrophysiology experiments, DMSO was included in the ACSF at a final concentration of 0.025% v/v. Hippocampal slices for immunoblotting were incubated in drug‐containing ACSF solution for 2 h, then stored at −80°C until use.

### 2.6 Extracellular Field Potential Recording

After at least two hours of incubation, slices were transferred into a recording chamber for electrophysiological measurements as previously described [19, 20, 21, 22] with continuous ACSF perfusion at 34°C. A bipolar stimulating electrode (MicroProbes, Gaithersburg, MD) was placed in the Schaffer collateral pathway. An extracellular recording pipette drawn with the PC‐10 Dual‐Stage Glass Micropipette Puller (Narishige, Amityville, NY) and filled with ACSF (2–6 MΩ) was placed in the stratum radiatum of CA1 to record field excitatory postsynaptic potentials (fEPSPs). For paired‐pulse facilitation (PPF), pairs of stimuli are separated by varying intervals. Ratios of fEPSP slopes from the second stimulus (fESP_2_) to fEPSP slopes from the first stimulus (fESP_1_) were calculated and plotted as a function of interstimulus intervals. For readily releasable pool (RRP) experiments, train stimulation (40 pulses, 10 ms interstimulus interval) was applied and fEPSP slopes from pulses 2–40 were normalized to the first pulse [23]. Basal synaptic transmission, represented by input–output responses, was determined as the slope of fEPSPs at various stimulus intensities and plotted as a function of fiber volley (FV) amplitude [23]. For LTP experiments, stimulus intensity was set at 50% of the amplitude at which the initial population spike appeared. LTP was induced after at least 10 min of stable baseline recording using a theta burst stimulation (TBS) protocol (10 bursts of stimuli, each of four pulses at 100 Hz, interburst interval of 200 ms, and 20s intervals between individual sweeps), and recording was continued for 60 min post‐TBS [22, 24, 25]. LTP was measured as an average of fEPSP slopes from 50 to 60 min after the end of induction and represented as the percentage increase from baseline. The data were recorded online using WinLTP software (University of Bristol, UK). Standard off‐line analyses of the data were conducted using Prism software (GraphPad Prism version 8, San Diego, California, USA).

### 2.7 Statistical Analysis

Statistical analysis was performed using GraphPad Prism 8 software. Normality was first assessed using the D'Agostino‐Pearson normality test and found to be normally distributed. Data were then analyzed by one‐way or two‐way analysis of variance (ANOVA), with or without repeated measures or mixed models where appropriate. Transgene (Tg) refers to 3xTg‐AD versus Control, while treatment (Tx) refers to the various drugs used for incubation. Unless otherwise noted, planned comparisons consisted of comparing Controls versus Controls treated with AdipoRon (Controls+AR), 3xTg‐AD mice, and 3xTg‐AD mice treated with AdipoRon (3xTg + AR), as well as comparing 3xTg‐AD versus 3xTg + AR. Results were considered significantly different when *p* < 0.05. Omnibus test results are described in the figure captions, and post hoc results are shown in the figures. All data are presented as means ± SEM.

## 3 Results

### 3.1 Altered Adiponectin and Adiponectin Receptor Expression in 3xTg‐AD Mice

**FIGURE 1:**
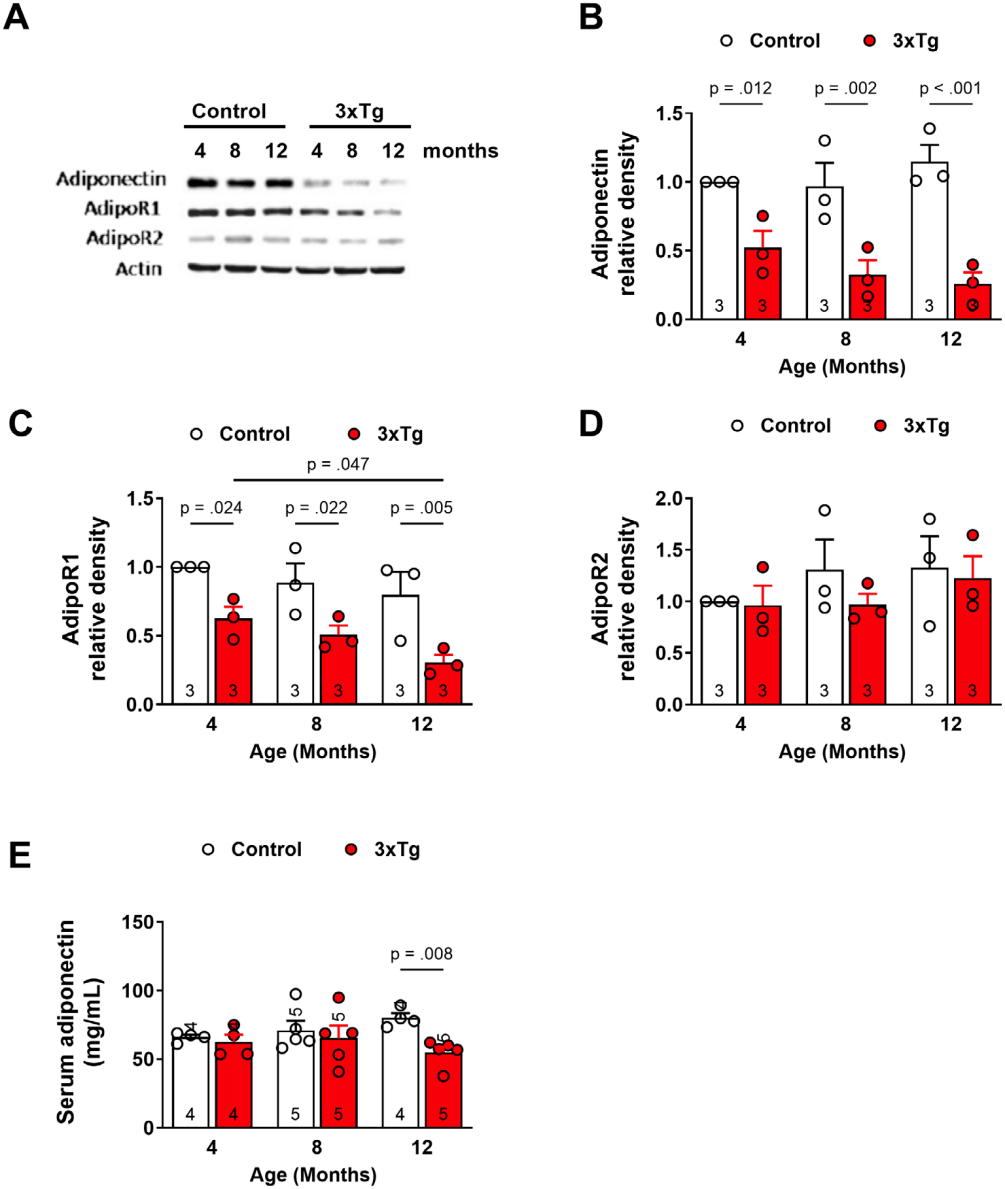
Altered adiponectin and adiponectin receptor levels in 3xTg‐AD mice. Hippocampal tissue (A–D) or serum (E) was isolated from 4, 8, and 12‐month‐old Control and 3xTg‐AD mice and used for Western blot or ELISA, respectively. (A) Representative immunoblots for graphs B–D. (B) Adiponectin/beta‐Actin relative expression. Two‐way RMANOVA: Tg*Age, [*F*(2, 12) = 1.67, *p* = 0.23]; Tg, [*F*(1, 12) = 52.27, *p* < 0.001]. (C) AdipoR1/beta‐Actin relative expression. Two‐way RMANOVA: Tg*Age, [*F*(2, 12) = 0.274, *p* = 0.82]; Tg, [*F*(1, 12) = 24.82, *p* < 0.001]. (D) AdipoR2/beta‐Actin relative expression. Two‐way RMANOVA: Tg*Age, [*F*(2, 12) = 0.279, *p* = 0.76]; Tg, [*F*(1, 12) = 0.85, *p* = 0.374]. (E) Serum adiponectin concentrations. Two‐way RMANOVA: Tg*Age, [*F*(2, 21) = 1.9, *p* = 0.173]; Tg, [*F*(1, 21) = 5.3, *p* = 0.031]. For Western blot, 40 μg of protein was loaded per lane. Symbols/bars represent mean ± SEM; for B–D, *n* = 3 mice per group; for E, *n* = 4–5 mice per group.

To evaluate whether hippocampal adiponectin or adiponectin receptor levels are altered in the 3xTg‐AD mice, we performed a Western blot on hippocampal tissue from 4, 8, and 12‐month‐old Control and 3xTg‐AD mice (Figure 1A). Total adiponectin expression was reduced in 3xTg‐AD hippocampi at 4, 8, and 12 months (Figure 1B). AdpoR1 hippocampal expression was also reduced in 3xTg‐AD mice at 4, 8, and 12 months (Figure 1C), whereas AdipoR2 hippocampal expression did not differ at any age examined (Figure 1D). Thus, there appears to be a reduction in the expression of adiponectin and AdipoR1, but not AdipoR2, in the hippocampus of 3xTg‐AD mice. To determine whether serum levels of adiponectin are altered in 3xTg‐AD mice, we performed ELISA for total adiponectin concentrations. Adiponectin serum levels were significantly lower in 12‐month 3xTg‐AD mice compared to Control mice (Figure 1E), suggesting that serum adiponectin levels are altered in aged 3xTg‐AD mice.

### 3.2 AdipoRon Rescues Deficits in Basal Synaptic Transmission and LTP Deficits in 3xTg‐AD Mice

**FIGURE 2:**
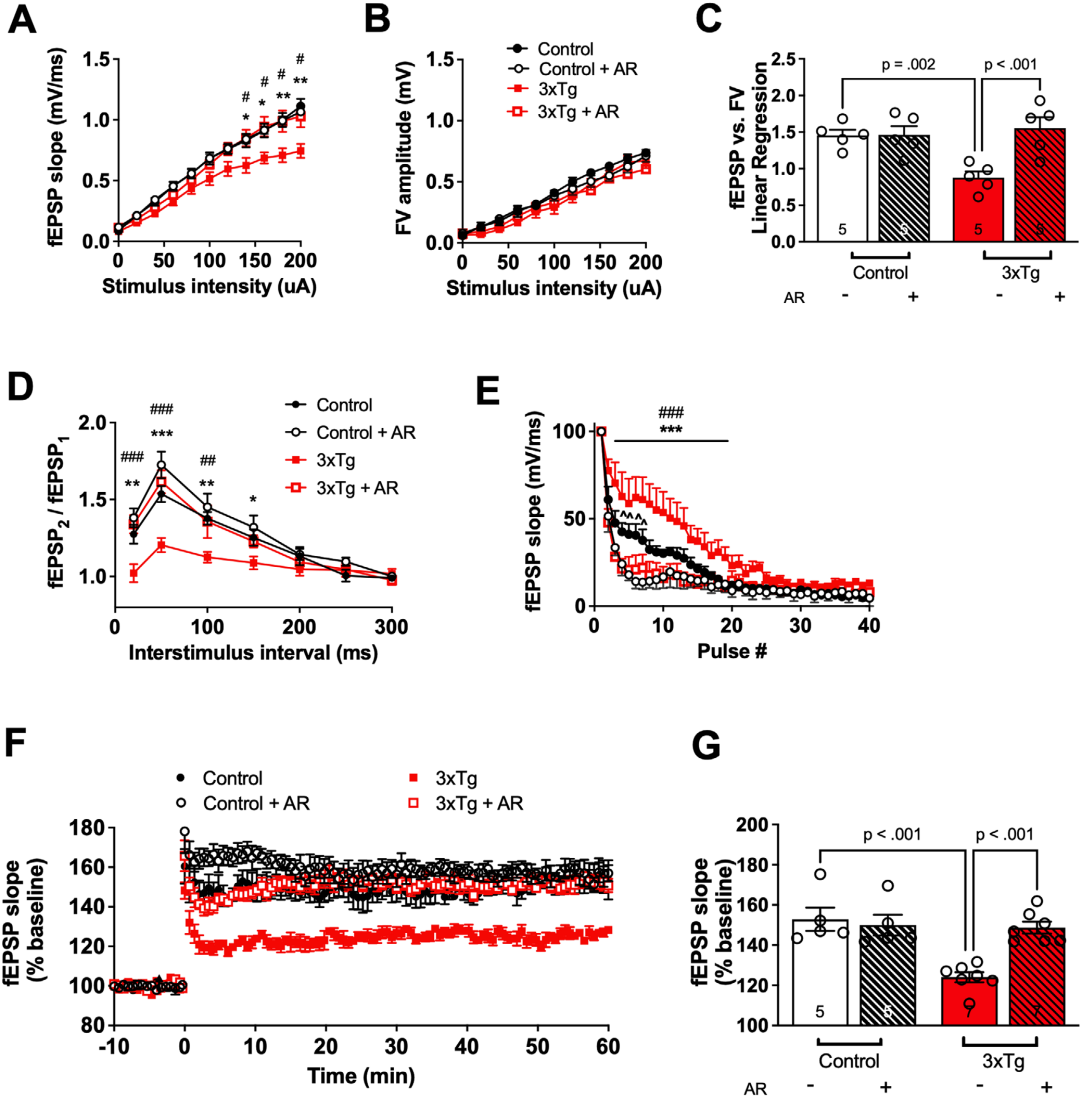
3xTg‐AD mice display deficits in basal synaptic transmission and LTP which are rescued by AdipoRon. Hippocampal slices were prepared from Control and 3xTg‐AD mice and incubated for 2‐h in ACSF‐drug solution prior to recording. (A) Input–output curve of fEPSP slope measured at increasing stimulus intensities. Two‐way RMANOVA: Tg*Tx*Intensity [*F*(10, 180) = 3.3, *p* < 0.001]. (B) Input–output curve of FV amplitude measured at increasing stimulus intensities. Two‐way RMANOVA: *p*s > 0.05. (C) Slope of the linear regression line of best fit from plotting fEPSP slope versus FV amplitude. Two‐way ANOVA: Tg*Tx [*F*(1, 16) = 9.0, *p* = 0.009]. (D) Paired‐pulse facilitation expressed as the ratio of the second stimulus fEPSP slope to the first stimulus fEPSP slope plotted as a function of interstimulus interval. Two‐way RMANOVA: Tg*Intensity [*F*(6, 120) = 2.9, *p* = 0.011] & Tx*Intensity [*F*(6, 120) = 5.1, < 0.001]. (E) Readily releasable pool expressed as the fEPSP slopes from stimuli 2–40 normalized to the first stimulus. Two‐way RMANOVA: Tx*Pulse [*F*(39, 741) = 9.3, *p* < 0.001]. (F) LTP graph represents fEPSP slope before and after induction by TBS. (G) LTP bar graph shows the average of fEPSPs 50–60 min following TBS induction normalized to baselines levels. Two‐way ANOVA: Tg*Tx [*F*(1, 20) = 12, *p* = 0.002]. Planned post hoc comparisons: (*) represents a significant difference in Control vs. 3xTg, (#) represents a significant difference in 3xTg vs. 3xTg + AR, (^) represents Control vs. Control+AR; symbols/bars represent mean ± SEM; *^/#/^^ *p* < 0.05, **/^##/^^^ *p* < 0.01; ***/^###/^^^^ *p* < 0.001; *n* = 5–6 slices from 4–5 mice per group.

To determine whether AdipoRon rescues deficits in hippocampal glutamatergic basal synaptic transmission in 3xTg‐AD mice, fEPSPs were measured at increasing stimulus intensities. We observed a divergence among the groups with increasing intensities (Figure 2A). Specifically, fEPSP slope was reduced in 3xTg‐AD mice compared to controls at 140, 160, 180, and 200 μA, indicating deficits in baseline glutamatergic synaptic transmission. The deficits in 3xTg‐AD mice were restored following incubation with AdipoRon. AdipoRon incubation had no effect at any stimulus intensity in the control group.

To determine whether the deficits in basal synaptic transmission in 3xTg‐AD mice are due to deficits in presynaptic axon recruitment, stimulus intensities versus FV amplitudes were compared and found to be similar between groups and treatments at all stimulus intensities (Figure 2B), suggesting that the changes in basal synaptic transmission are not due to changes in axon recruitment. Evaluation of the FV amplitude versus fEPSP slope provides a measure of basal synaptic transmission while controlling for axon recruitment and, thus, is the ideal measure of the overall basal synaptic transmission [26]. The linear regression slope was significantly reduced in the 3xTg‐AD group but restored to that of controls following treatment with AdipoRon (Figure 2C). The increase in synaptic transmission by AdipoRon incubation in the 3xTg‐AD group, but not the Control group, indicates a selective improvement in glutamatergic neurotransmission in AD pathology.

To determine whether alterations in basal synaptic transmission could be related to alterations in presynaptic glutamate availability, we evaluated PPF and depletion of the RRP. There was a significant reduction in PPF at 20, 50, 100, and 150 ms in 3xTg‐AD mice compared to controls (Figure 2D). Notably, AdipoRon treatment significantly increased PPF in 3xTg‐AD mice to a level not significantly different from controls. As there is an inverse relationship between PPF and presynaptic release probability, this indicates that 3xTg‐AD mice have an increased presynaptic release probability and that AdipoRon leads to a reduction in presynaptic release probability.

RRP represents glutamatergic synaptic vesicles available for immediate release. The depletion of the RRP was significantly slower in 3xTg‐AD mice compared to controls, indicating a larger size of the RRP of glutamate in presynaptic hippocampal neurons in 3xTg‐AD mice (Figure 2E). AdipoRon led to a more rapid depletion of the RRP in both 3xTg‐AD and control groups. Taken together, this indicates that 3xTg‐AD mice have a higher level of presynaptic glutamate availability, which may indicate an increased propensity for glutamatergic excitotoxicity, and AdipoRon reduces glutamate availability in both 3xTg‐AD and control mice. Thus, the actions of AdipoRon on presynaptic parameters do not appear to be selective for AD pathology.

To determine whether AdipoRon incubation rescues deficits in synaptic plasticity in 3xTg‐AD mice, we measured LTP in hippocampal slices. Hippocampal LTP is correlated with performance on hippocampal‐based cognitive tests, and thus, LTP has been termed the cellular correlate of learning and memory [27]. LTP was reduced in the 3xTg‐AD mice compared to controls, and AdipoRon incubation increased LTP in treated 3xTg‐AD mice to that of controls (Figure 2F,G).

### 3.3 AdipoRon Incubation Alters Phosphorylation of AMPK, GSK3β, and GluA1 in 3xTg‐AD Hippocampal Slices

**FIGURE 3:**
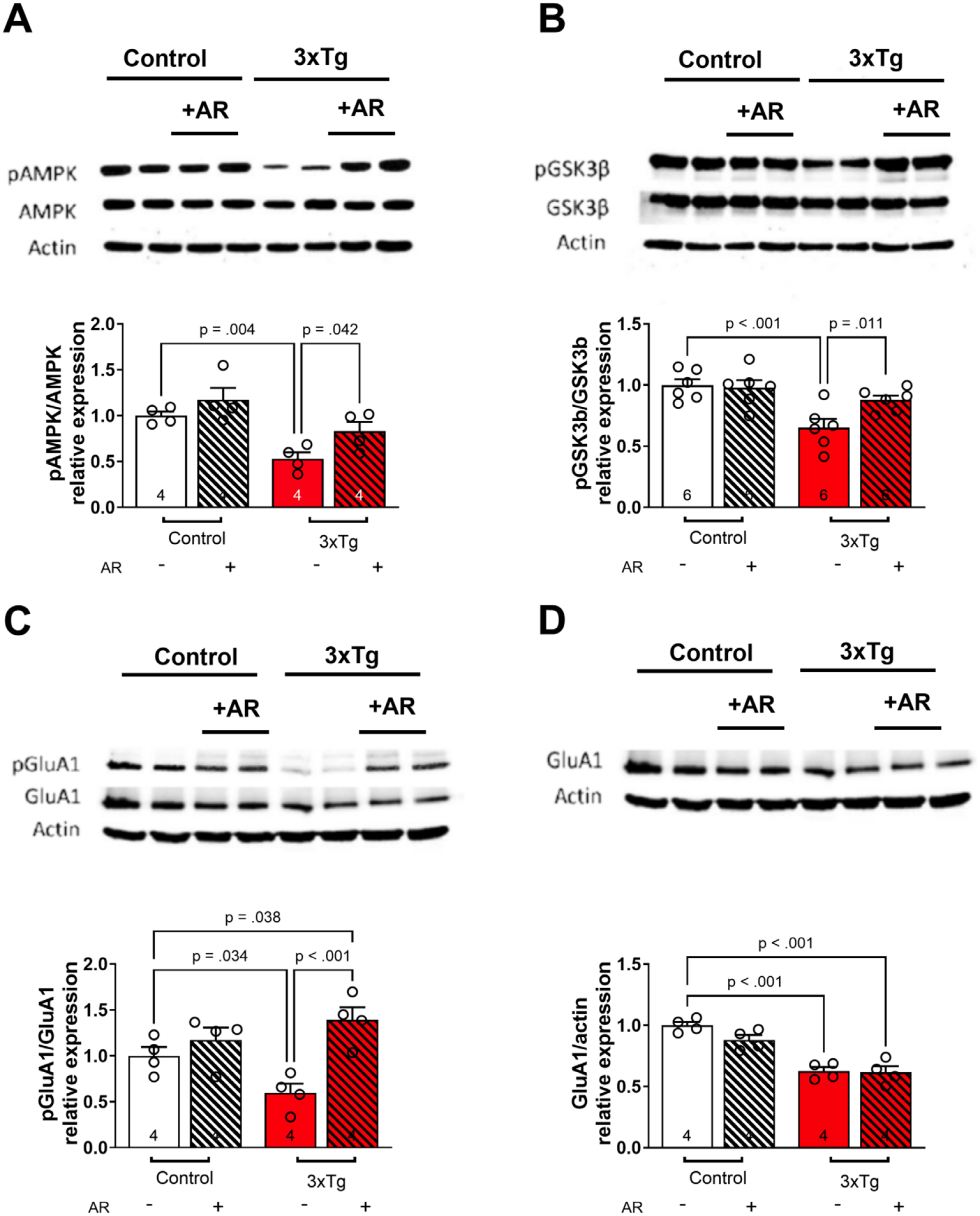
Adiporon incubation increases pAMPK, pGSK3β, and pGluR1 Ser831 levels in hippocampal slices from 3xTg‐AD mice. Hippocampal slices were prepared from Control and 3xTg‐AD mice and incubated for 2‐h in ACSF‐drug solution. Then, hippocampal tissue was isolated and kept at −80°C until use. Representative immunoblots from hippocampal lysate: (A) pAMPK/AMPK relative expression ratio. Two‐way ANOVA: Tg [*F*(1, 12) = 19, *p* < 0.001] & Tx [*F*(1, 12) = 6.4, *p* = 0.026]. (B) pGSK3β/GSK3β relative expression ratio. Two‐way ANOVA: Tg*Tx [*F*(1, 20) = 4.7, *p* = 0.042]. (C) pGluA1 Ser831/GluA1 relative expression ratio. Two‐way ANOVA: Tg*Tx [*F*(1, 12) = 6.9, *p* = 0.022]. (D) GluA1/beta‐Actin relative expression ratio. Two‐way ANOVA: Tg [*F*(1, 12) = 68, *p* < 0.001]. 40 μg of protein was loaded per lane. *n* = 4–6 mice per group.

To investigate signaling mechanisms associated with synaptic alterations following AdipoRon incubation, we performed Western blot analysis following hippocampal slice incubation. We first evaluated AMPK, a major downstream signaling molecule for AdipoR1 [28, 29, 30]. 3xTg‐AD mice exhibited reduced pAMPK that was restored to that of controls following treatment with AdipoRon (Figure 3A). Next, we evaluated GSK3β, a key player in the pathophysiology of AD [31], which is phosphorylated and thus inactivated following activation of adiponectin receptors [8]. pGSK3β was decreased in 3xTg‐AD mice and restored to that of controls by treatment with AdipoRon (Figure 3B). This suggests that AdipoRon incubation may improve synaptic parameters via activation of AMPK and inhibition of GSK3β.

Next, we considered whether AdipoRon incubation alters the phosphorylation of glutamatergic receptor subunits involved in the maintenance of LTP. GluA1 is a subunit of the glutamatergic α‐amino‐3‐hydroxy‐5‐methyl‐4‐isoxazolepropionic acid receptor (AMPAR), and an increase in phosphorylation of Ser831 increases the conductance of the AMPARs and thus may enhance basal synaptic transmission and LTP [32]. 3xTg‐AD mice exhibited reduced GluA1 Ser831, and AdipoRon treatment increased GluA1 Ser831 phosphorylation in 3xTg‐AD mice (Figure 3C). This improvement in GluA1 Ser831 occurred despite a failure of AdipoRon to restore the deficit in GluA1 receptor levels observed in 3xTg‐AD mice (Figure 3D). Thus, AdipoRon incubation may be enhancing basal synaptic transmission and LTP in part via increased phosphorylation of GluA1 Ser831.

### 3.4 An AMPK Inhibitor Prevents AdipoRon‐Induced Enhancement in LTP but Not Presynaptic Measures in 3xTg‐AD Hippocampal Slices

Because AdipoRon treatment increased phosphorylation of AMPK, and AMPK is an important mediator of synaptic function [18], we hypothesized that an AMPK inhibitor may prevent AdipoRon‐induced alterations in synaptic function in 3xTg‐AD mice. We utilized the AMPK inhibitor Compound C (CC) to test this hypothesis, and we also utilized AICAR, an AMPK activator, to serve as a positive control.

**FIGURE 4:**
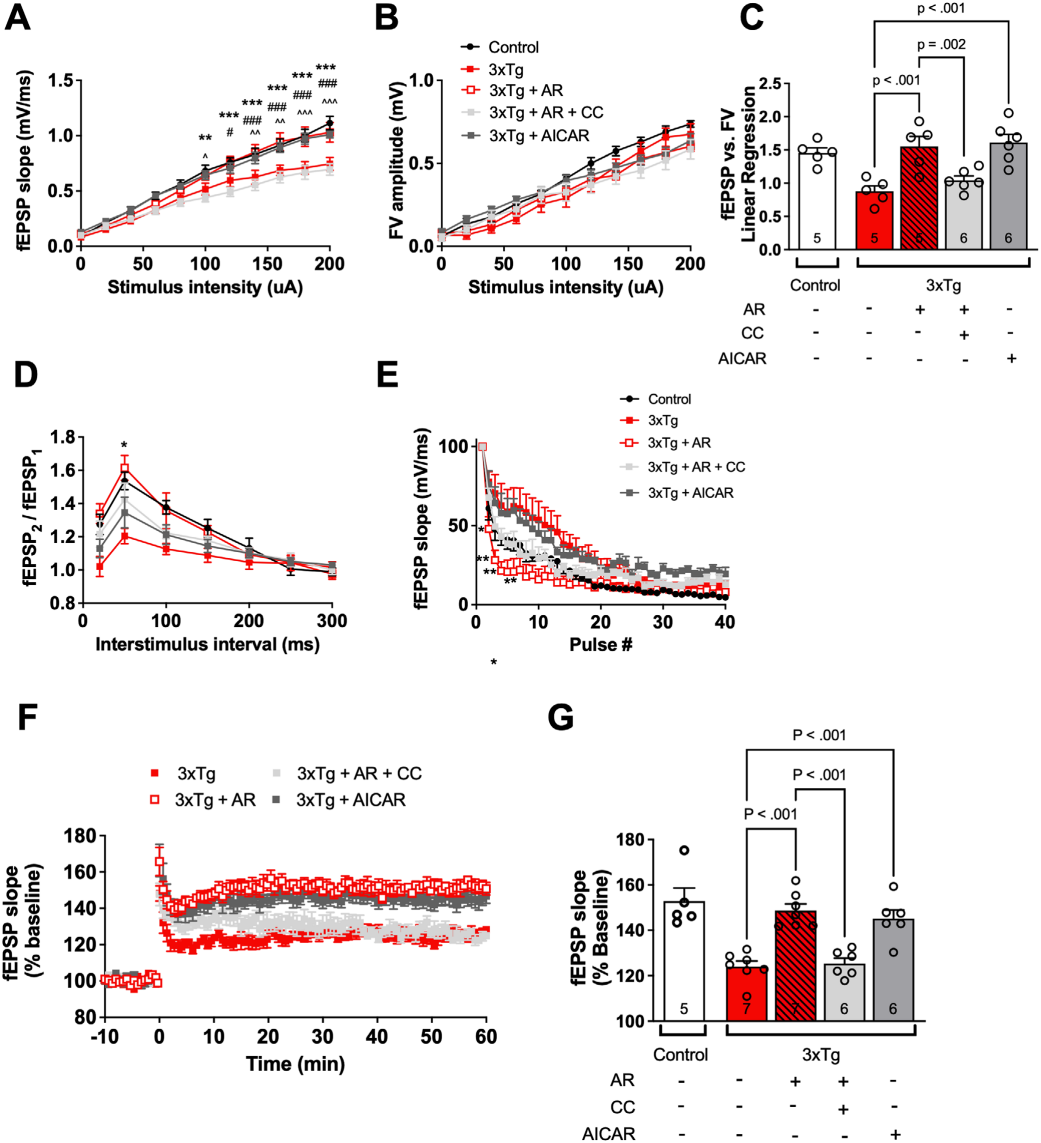
AdipoRon fails to increase basal synaptic transmission and LTP in 3xTg‐AD mice in the presence of an AMPK inhibitor. Hippocampal slices were prepared from controls and 3xTg‐AD mice and incubated for 2 h in ACSF‐drug solution prior to recording. (A) Input–output curve of fEPSP slope measured at increasing stimulus intensities. One‐way RMANOVA: Tx*Intensity [*F*(30, 200) = 4.2, *p* < 0.001]. (B) Input–output curve of FV amplitude measured at increasing stimulus intensities. One‐way RMANOVA: Tx*Intensity [*F*(30, 200) = 0.49, *p* = 0.695]. (C) Slope of the linear regression line of best fit from plotting fEPSP slope versus FV amplitude. One‐way ANOVA: Tx [*F*(4, 22) = 9.8, *p* < 0.001] in 3xTg mice. (D) Paired‐pulse facilitation expressed as the ratio of the second stimulus fEPSP slope to the first stimulus fEPSP slope plotted as a function of interstimulus interval. One‐way RMANOVA: Tx*Interval [*F*(18, 210) = 2.8, *p* < 0.001]. (E) Readily releasable pool expressed as the fEPSP slopes from stimuli 2–40 normalized to the first stimulus. One‐way RMANOVA: Tx*Pulse [*F*(117, 741) = 2.9, *p* < 0.001]. (F) LTP graph represents fEPSP slope before and after induction by TBS in 3xTg mice. (G) LTP bar graph shows the average of fEPSPs 50–60 min following TBS induction normalized to baseline levels. One‐way ANOVA: Tx [*F*(3, 22) = 19.61, *p* < 0.001]. For (A–G), the Control group is shown for visual comparison but was not included in analyses or planned post hoc comparisons, which included a significant difference in 3xTg versus 3xTg + AR (#), 3xTg + AR versus 3xTg + AR + CC (*) and 3xTg versus 3xTg + AICAR (^); *^/#/^^ *p* < 0.05, **/^##/^^^ *p* < 0.01; ***/^###/^^^^ *p* < 0.001; Symbols/bars represent mean ± SEM; *n* = 5–7 from 4–5 mice per group.

First, we evaluated the ability of AdipoRon to enhance basal synaptic transmission in the presence of CC in 3xTg‐AD mice. In the presence of CC, AdipoRon failed to enhance basal synaptic transmission, whereas the AMPK activator, AICAR, significantly enhanced basal synaptic transmission at higher stimulus intensities in 3xTg‐AD mice (Figure 4A). As expected, there were no significant differences among the groups for FV analysis (Figure 4B). We next examined FV amplitude versus fEPSP slope analysis for overall changes in basal synaptic transmission (Figure 4C). Again, AdipoRon failed to enhance basal synaptic transmission in the presence of CC, while AICAR increased basal synaptic transmission in 3xTg‐AD mice. This provides evidence that AdipoRon may enhance basal synaptic transmission in 3xTg‐AD mice via activation of AMPK. In contrast, we observed no effect of any of the treatments in control hippocampal slices for fEPSP, FV amplitude, or fEPSP slope versus FV amplitude (Figure S1A–C, respectively).

We next examined the role of AMPK in mediating AdipoRon effects on the presynaptic parameters, PPF and RRP. For PPF in 3xTg‐AD mice, AdipoRon only marginally increased PPF in the presence of CC, and AICAR failed to increase PPF at any interstimulus intensity (Figure 4D). Similarly, for RRP, although AdipoRon was able to somewhat enhance the initial depletion of the RRP in the presence of CC (pulses 2–6), it was less effective compared to AdipoRon alone, and there was no significant change following AICAR incubation (Figure 4E). As AMPK activation alone was insufficient to increase PPF or increase the depletion of RRP, AMPK does not appear to be the major mechanism by which AdipoRon alters presynaptic parameters. However, since AdipoRon was unable to significantly increase PPF or significantly enhance the depletion of the RRP in the presence of the AMPK inhibitor CC, AMPK may be contributing to some of the activity of AdipoRon. In control slices, manipulation of AMPK had no effect on PPF (Figure S1D), whereas for RRP, AdipoRon maintained the ability to significantly enhance depletion of the RRP in the presence of CC, and AICAR did not significantly alter the RRP (Figure S1E). This suggests that AdipoRon is altering RRP by a mechanism that is not dependent on AMPK activation in control hippocampal slices.

Next, we determined whether AdipoRon alters synaptic plasticity in 3xTg‐AD hippocampal slices in the presence of an AMPK inhibitor. For 3xTg‐AD mice, AdipoRon was unable to enhance LTP in the presence of the AMPK inhibitor CC, whereas the AMPK activator, AICAR, significantly enhanced LTP (Figure 4F,G). To determine whether inhibition or activation of AMPK might alter LTP in Control hippocampal slices, we compared the effects of drug incubation and found no significant differences among groups (Figure S1F,G). Thus, in 3xTg‐AD hippocampal slices, AMPK activation or AdipoRon significantly enhances LTP, and AdipoRon is unable to enhance LTP in the presence of an AMPK inhibitor. This implies that AdipoRon may be enhancing LTP via a mechanism dependent on AMPK.

### 3.5 Inhibition of AMPK Blocks AdipoRon‐Induced Increased Phosphorylation of GSK3β and GluA1

**FIGURE 5:**
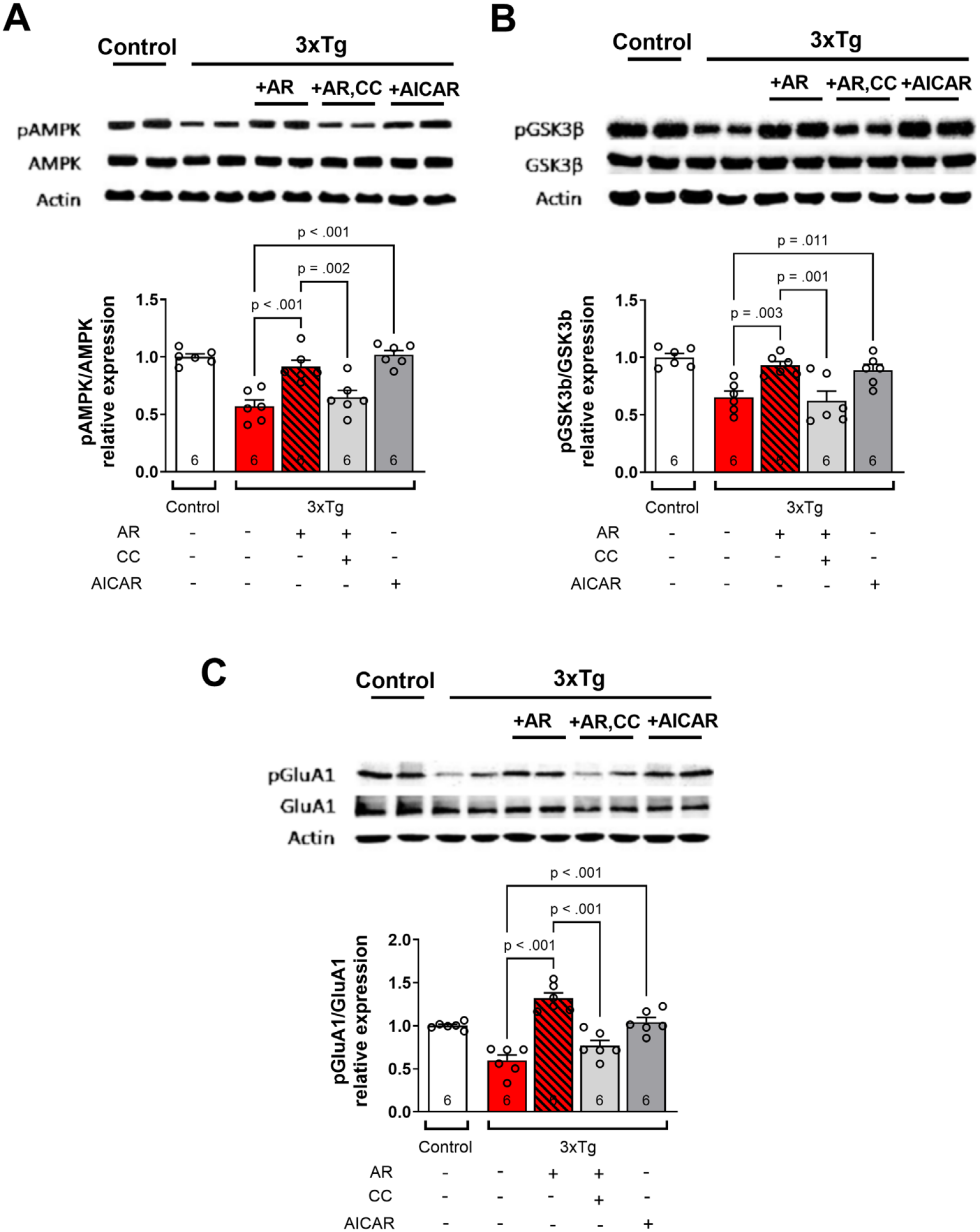
AdipoRon fails to increase pAMPK, pGSK3b, and pGluA1 in 3xTg‐AD hippocampal slices in the presence of an AMPK inhibitor. Hippocampal slices were prepared from Control and 3xTg mice and incubated for 2 h in ACSF‐drug solution. Then, hippocampal tissue was isolated and kept at −80°C until use. Representative immunoblots of 40 μg of hippocampal lysate per lane showing: (A) pAMPK/AMPK relative expression ratio. One‐way ANOVA: [*F*(3, 20) = 15.95, *p* < 0.001]. (B) pGSK3β/GSK3β relative expression ratio. One‐way ANOVA: [*F*(3, 20) = 7.178, *p* = 0.002]. (C) pGluA1 Ser831/GluA1 relative expression ratio. One‐way ANOVA: [*F*(3, 20) = 26.27, *p* < 0.001]. For (A–C), the Control group is shown for visual comparison but was not included in analyses or planned post hoc comparisons. Symbols/bars represent mean ± SEM; *n* = 6 mice per group.

To determine whether the use of an AMPK inhibitor could prevent the AdipoRon‐induced enhancement of GSK3β and GluA1 phosphorylation in 3xTg‐AD hippocampal slices, we performed a Western blot following slice incubation. First, we confirmed the prevention of AdipoRon‐induced AMPK phosphorylation in the presence of CC, as well as phosphorylation of AMPK via AICAR. CC prevented an AdipoRon‐induced increase in AMPK phosphorylation, while AICAR enhanced the phosphorylation of AMPK (Figure 5A).

Next, we evaluated the ability of CC to prevent the AdipoRon‐induced increase in phosphorylation of GSK3β. The increase in phosphorylation of GSK3β was prevented by CC incubation, and AICAR increased the phosphorylation of GSK3β in 3xTg‐AD hippocampal slices (Figure 5B). This implies that AdipoRon‐induced enhancement of GSK3β phosphorylation and, thus, inactivation of GSK3β are dependent on activation of AMPK.

We next determined whether enhancement of GluA1 phosphorylation following AdipoRon incubation is also dependent on AMPK activation. Phosphorylation of GluA1 by AdipoRon incubation was prevented by co‐incubation in CC, whereas AICAR alone was able to enhance phosphorylation of GluA1 (Figure 5C). Thus, the ability of AdipoRon to enhance Ser9 phosphorylation of GSK3β and Ser831 phosphorylation of GluA1 appears to be dependent on the activation of AMPK.

## 4 Discussion

These results provide evidence for dysregulation of adiponectin in AD and indicate that adiponectin receptor signaling may reduce AD pathology by directly influencing synaptic function. Furthermore, we have identified AMPK as a critical player for adiponectin receptor signaling in rescuing specific synaptic deficits. In the 3xTg‐AD mice, we found a reduction in hippocampal adiponectin expression across various ages and altered AdipoR1 expression, without significant changes in AdipoR2. Overall, our data suggest that there may be a progressive dysregulation of adiponectin in the 3xTg‐AD model.

To determine whether activation of adiponectin receptors ameliorates synaptic deficits, we first evaluated the influence of AdipoRon on basal synaptic transmission. Deficits in basal synaptic transmission are established to occur in various animal models of AD, and deficits correlate with the progression of the disease [33, 34, 35]. Basal synaptic transmission is influenced by many factors, including the release of glutamate, glutamate uptake, and the expression or functionality of glutamatergic receptors, specifically AMPARs [26]. In the current study, AdipoRon enhanced the phosphorylation of the GluA1 subunit of AMPAR at Ser831 in the 3xTg‐AD group, which is associated with increased conductance of the receptor [36]. This could be one reason for enhanced basal synaptic transmission in 3xTg mice following AdipoRon incubation and may also explain the lack of change in basal synaptic transmission for the control mice exposed to AdipoRon, as AMPAR phosphorylation was unchanged in controls following AdipoRon treatment. A study showed an increase in surface expression of GluA1 following adiponectin slice incubation [15], which implies that adiponectin receptor signaling alters AMPAR trafficking in addition to influencing AMPAR activity. Therefore, it seems likely that adiponectin receptor signaling enhances basal synaptic transmission in part by influencing AMPARs. Interestingly, AdipoRon enhanced the depletion of presynaptic glutamate and reduced the presynaptic release probability of glutamate, which could decrease basal synaptic transmission in the absence of other factors. Thus, the rescue of basal synaptic transmission by AdipoRon appears unrelated to presynaptic alterations.

In the current study, the AdipoRon‐induced increase in basal synaptic transmission and GluA1 phosphorylation was inhibited by co‐incubation with the AMPK inhibitor, CC. This indicates that activation of AMPK is required for both of these effects. Interestingly, incubation in AdipoRon did not significantly enhance phosphorylation of AMPK, phosphorylation of GluA1, or basal synaptic transmission in hippocampal slices from the Control group. We hypothesize that the normalization of basal synaptic transmission following AdipoRon incubation of 3xTg‐AD hippocampal slices is due to the restoration of normal postsynaptic adiponectin receptor signaling and that a further enhancement of postsynaptic adiponectin receptor signaling does not yield further augmentation.

In the current study, we observed an increase in presynaptic release probability (reduction in PPF) along with a slower depletion of presynaptic glutamate in the 3xTg‐AD mice. This implies increased presynaptic glutamate availability, suggesting an increased potential for glutamatergic excitotoxicity [37]. Elevated glutamate release has been associated with the progression of both Aβ and tau pathology, in addition to excitotoxicity [as reviewed in 38, 39]. For example, studies indicate that glutamate release prompts tau, an intracellular protein, to enter the extracellular space [40, 41, 42], where it can then be taken by nearby neurons, leading to aggregation with native tau in a prion‐like manner [40, 43]. This process precedes synaptic and neuronal loss and is corroborated by tau PET imaging in human AD patients [44]. Moreover, hippocampal hyperactivity has also been observed in individuals with mild cognitive impairment [45] and those with a genetic or familial predisposition to AD [46, 47]. In animal models of AD, there are differing findings regarding the influence of AD pathology on presynaptic glutamate release probability, which may be time‐dependent [13]. Some have proposed a two‐stage model in which increased glutamatergic availability and glutamatergic hyperexcitability occur initially, followed by glutamatergic hypoactivity [48, 49]. However, paradoxically, increased glutamate availability may co‐exist with impaired synaptic transmission [13, 34], which is observed in the current study. The increase in presynaptic glutamate release and availability in the 3xTg‐AD group was reduced by AdipoRon incubation, and AdipoRon also reduced presynaptic glutamate availability in the Control group. Our data suggest that activation of AMPK is not the primary mechanism by which AdipoRon influences presynaptic parameters, but inhibition of AMPK may alter the presynaptic effects of AdipoRon.

The mechanism by which adiponectin receptor signaling alters presynaptic parameters is unclear from the current study. However, we previously found increased expression of the vesicular glutamate transporter (VGLUT) in adiponectin knockout mice [50]. VGLUT is responsible for the packaging of glutamate into presynaptic vesicles for eventual release, and an increase in VGLUT is correlated with enhanced presynaptic glutamate availability [51, 52]. Thus, adiponectin receptor signaling may lead to alterations in glutamate availability by influencing presynaptic glutamatergic vesicles. Modulation of presynaptic parameters through adiponectin receptor signaling may reduce AD pathology by reducing excitotoxicity and limiting the propagation of Aβ. However, additional studies are needed to determine whether these presynaptic changes contribute to improved synaptic function in AD.

Deficits in LTP are an integral component of the synaptic dysfunction observed in AD pathology [53]. In the current study, we observed that AdipoRon enhances LTP in the hippocampal Schaffer collateral pathway in the 3xTg‐AD mice without affecting control LTP. Other groups have also reported an influence of adiponectin receptor signaling on LTP. Adiponectin‐mediated enhancement of LTP in vivo has been shown in the hippocampal perforant pathway of the hippocampus in adult rats [14]. The effects of adiponectin receptor signaling on LTP may be dose‐dependent, as we previously found that AdipoRon reduced LTP in the hippocampal Schaffer collateral pathway in control mice at higher concentrations [50]. Moreover, adiponectin incubation of hippocampal slices in the 5xFAD model of AD enhances LTP in the Schaffer collateral pathway [15]. Thus, adiponectin receptor signaling appears to enhance LTP in models with amyloid pathology (e.g., the 5xFAD model), as well as in the 3xTg‐AD model with both amyloid and tau pathology [16], an effect we demonstrate is AMPK‐dependent. We found that inhibition of AMPK prevents AdipoRon‐mediated enhancement of LTP, and that the AMPK activator, AICAR, also enhances LTP in the 3xTg‐AD hippocampal slices. This was supported by a reduction of hippocampal pAMPK in the 3xTg‐AD, which was normalized following incubation in AdipoRon. Thus, it appears that activation of AMPK is required for adiponectin receptor signaling enhancement of LTP. In addition to increased activation of AMPK, AdipoRon incubation increased phosphorylation and thus inactivation of GSK3β, an effect that was absent in the presence of the AMPK inhibitor. Importantly, AMPK‐induced inactivation of GSK3β via Ser9 phosphorylation promotes LTP in vivo [54]. GSK3β is a major mediator of synaptic plasticity, as inhibition of GSK3β promotes LTP while activation of GSK3β promotes long‐term depression (LTD) [55, 56]. Additionally, GSK3β inactivation is associated with enhanced Ser831 phosphorylation of GluA1 [57], which may promote LTP via enhanced conductance of AMPARs. Thus, the AdipoRon‐induced inactivation of GSK3β, along with increased conductance of AMPARs, is a potential mechanism by which AdipoRon enhanced LTP in the current study.

Some limitations to our study include a lack of behavioral data and the exclusion of male mice. While our data demonstrate rescue of synaptic function by AdipoRon, the present study did not evaluate whether these electrophysiological improvements translate into measurable cognitive benefits. However, prior studies have reported that AdipoRon treatment improved spatial memory in AD mouse models [58, 59], which aligns with the improvement in synaptic plasticity found in the current study. Another limitation of our study is the use of only female 3xTg‐AD mice, because male mice of this strain typically display an attenuated phenotype. While this approach ensured that our experimental cohort exhibited robust AD‐related pathology, it may limit the generalizability of our findings to male subjects. One study using both male and female 5xFAD mice found a reduction in neuronal AdipoR1 expression [60]; however, it remains unclear whether sex‐based differences are present for adiponectin signaling dysregulation. Future work should investigate the extent to which adiponectin signaling changes are model‐specific, sex‐dependent, or a generalizable feature of AD pathology. Additionally, further investigation into the dose‐dependent and long‐term effects of AdipoRon is warranted.

## 5 Conclusion

In summary, the key findings from the current study are that the adiponectin receptor agonist AdipoRon rescues basal synaptic transmission and LTP in the Schaffer collateral pathway of the hippocampus in 3xTg‐AD mice through a mechanism that appears to be dependent on the activation of AMPK. AdipoRon also alters presynaptic glutamate availability, but this does not appear to be solely dependent on the activation of AMPK. AdipoRon enhances Ser9 phosphorylation of GSK3β and Ser831 phosphorylation of GluA1, and these effects are blocked by inhibition of AMPK. Thus, we hypothesize that AdipoRon‐mediated improvement of basal synaptic transmission and LTP is related to increased conductance of GluA1‐containing AMPARs and inactivation of GSK3β following activation of AMPK. The current study provides evidence that adiponectin receptor signaling may directly reduce synaptic dysfunction in AD and contributes to the growing evidence for the use of adiponectin receptor agonists in the treatment of AD.

## Author Contributions

J.B. and P.D.P. performed experiments. J.B., P.D.P., and M.N.R. analyzed data. J.B., V.S., and M.N.R. conceptualized and designed experiments. J.B. and M.N.R. wrote the manuscript. M.N.R. and V.S. oversaw experimentation and analysis. J.B. and V.S. provided funding for the experiments.

## Ethics Statement

This study was approved by the Institutional Animal Care and Use Committee of Auburn University in compliance with the Guide for the Care and Use of Laboratory Animals (National Research Council, 2011).

## Conflicts of Interest

The authors declare no conflicts of interest.

## Supporting information


**Figure S1:** Modulation of AMPK does not alter basal synaptic transmission or LTP in Control mice. Hippocampal slices were prepared from controls and 3xTg mice and incubated for 2‐h in ACSF‐drug solution prior to recording. (A) Input–output curve of fEPSP slope measured at increasing stimulus intensities in control mice. One‐way RMANOVA: Tx*Intensity, [*F*(30, 180) = 1.4, *p* = 0.095]. (B) Input–output curve of fiber volley (FV) amplitude measured at increasing stimulus intensities in control mice. One‐way RMANOVA: Tx*Intensity, [*F*(30, 180) = 1.5, *p* = 0.053]. (C) Slope of the linear regression line of best fit from plotting fEPSP slope versus FV amplitude for controls. One‐way ANOVA: Tx, [*F*(3, 18) = 0.05, *p* = 0.984]. (D) Paired‐pulse facilitation expressed as the ratio of the second stimulus fEPSP slope to the first stimulus fEPSP slope plotted as a function of interstimulus interval in controls. One‐way RMANOVA: Tx*Interval, [*F*(24, 150) = 0.88, *p* = 0.635]. (E) Readily releasable pool expressed as the fEPSP slopes from stimuli 2–40 normalized to the first stimulus in controls. (F) LTP graph represents fEPSP slope before and after induction by theta burst stimulation (TBS) in control mice. (G) LTP bar graph shows the average of fEPSPs recorded during the time period 50–60 min following TBS induction, normalized to baseline levels in control mice. One‐way ANOVA: Tx, [*F*(3, 18) = 0.92, *p* = 0.449].Symbols/bars represent mean ± SEM; *n* = 5–7 slices from 4 to 5 mice per group.

## Data Availability

The data that support the findings of this study are available on request from the corresponding authors.
