# Supplementary material for: Adiponectin Receptor Agonist Ameliorates Synaptic Dysfunction in 3xTg Alzheimer's Disease Mouse Model by Activation of AMPK
Source: CNS Neurosci Ther. 2025 Sep 22;31(9):e70616. doi: 10.1111/cns.70616 (PMC12454683; doi:10.1111/cns.70616)
Supplement: Supplementary file 1 — Figure S1: Modulation of AMPK does not alter basal synaptic transmission or LTP in Control mice. Hippocampal slices were prepared from controls and 3xTg mice and incubated for 2‐h in ACSF‐drug solution prior to recording. (A) Input–output curve of fEPSP slope measured at increasing stimulus intensities in control mice. One‐way RMANOVA: Tx*Intensity, [F(30, 180) = 1.4, p = 0.095]. (B) Input–output curve of fiber volley (FV) amplitude measured at increasing stimulus intensities in control mice. One‐way RMANOVA: Tx*Intensity, [F(30, 180) = 1.5, p = 0.053]. (C) Slope of the linear regression line of best fit from plotting fEPSP slope versus FV amplitude for controls. One‐way ANOVA: Tx, [F(3, 18) = 0.05, p = 0.984]. (D) Paired‐pulse facilitation expressed as the ratio of the second stimulus fEPSP slope to the first stimulus fEPSP slope plotted as a function of interstimulus interval in controls. One‐way RMANOVA: Tx*Interval, [F(24, 150) = 0.88, p = 0.635]. (E) Readily releasable pool expressed as the fEPSP slopes from stimuli 2–40 normalized to the first stimulus in controls. (F) LTP graph represents fEPSP slope before and after induction by theta burst stimulation (TBS) in control mice. (G) LTP bar graph shows the average of fEPSPs recorded during the time period 50–60 min following TBS induction, normalized to baseline levels in control mice. One‐way ANOVA: Tx, [F(3, 18) = 0.92, p = 0.449].Symbols/bars represent mean ± SEM; n = 5–7 slices from 4 to 5 mice per group. [file CNS-31-e70616-s001.docx]

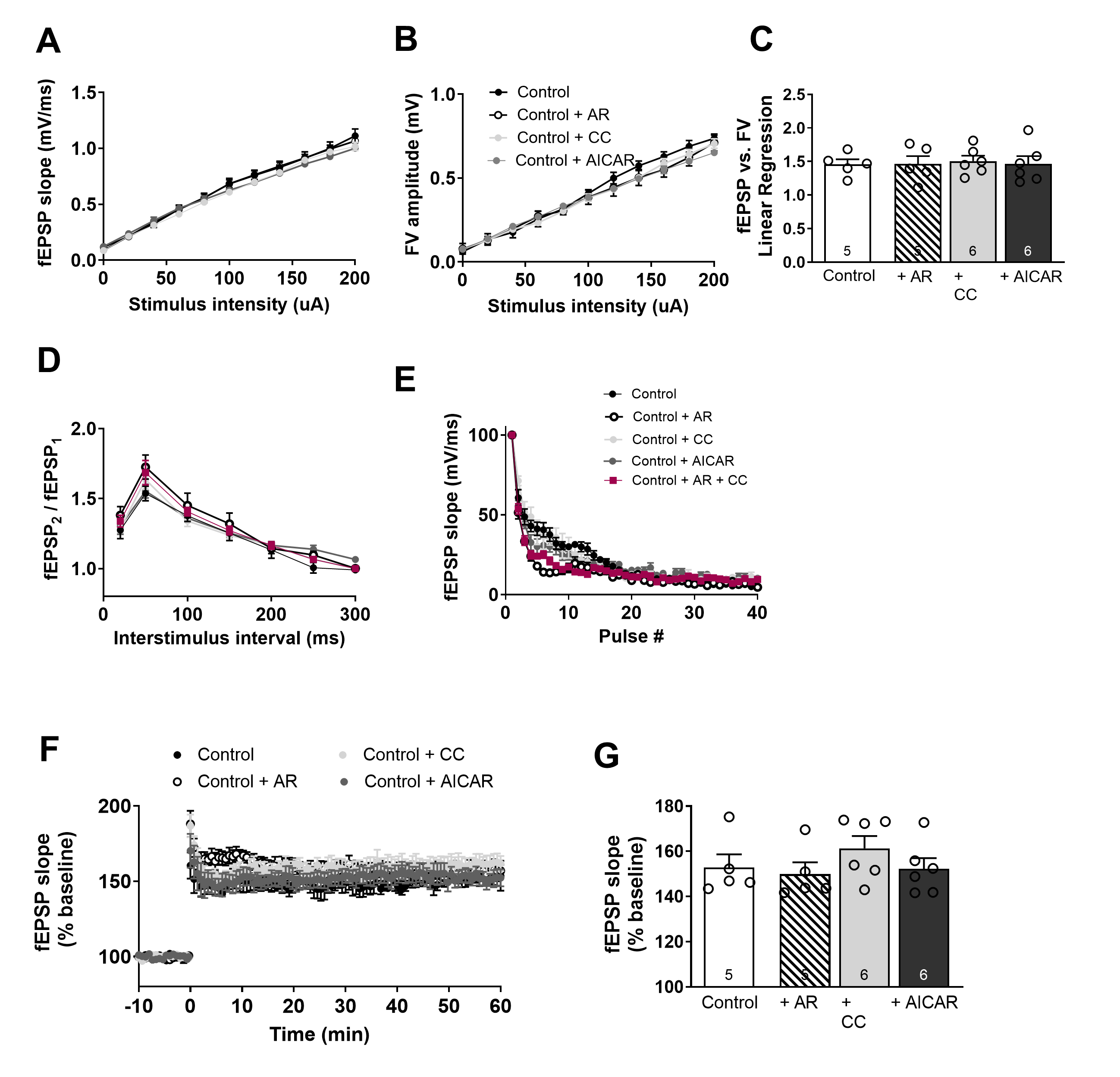


***Supplementary Figure 1. Modulation of AMPK does not alter basal synaptic transmission or LTP in Control mice.*** *Hippocampal slices were prepared from controls and 3xTg mice and incubated for 2-hours in ACSF-drug solution prior to recording. (A) Input-output curve of fEPSP slope measured at increasing stimulus intensities in control mice. One-way RMANOVA: Tx*Intensity, [F(30,180)=1.4, p=.095]. (B) Input-output curve of FV amplitude measured at increasing stimulus intensities in control mice. One-way RMANOVA: Tx*Intensity, [F(30,180)=1.5, p=.053]. (C) Slope of the linear regression line of best fit from plotting fEPSP slope versus FV amplitude for controls. One-way ANOVA: Tx, [F(3,18)=0.05, p=.984]. (D) Paired-pulse facilitation expressed as the ratio of the second stimulus fEPSP slope to the first stimulus fEPSP slope plotted as a function of interstimulus interval in controls. One-way RMANOVA: Tx*Interval, [F(24,150 =0.88, p=.635]. (E) Readily-releasable pool expressed as the fEPSP slopes from stimuli 2-40 normalized to the first stimulus in controls.* *(F) LTP graph represents fEPSP slope before and after induction by TBS in control mice. (G) LTP bar graph shows the average of fEPSPs recorded during the time period 50-60 min following TBS induction normalized to baselines levels in control mice. One-way ANOVA: Tx, [F(3,18)=0.92, p=.449].Symbols/bars represent mean ± SEM; n = 5-7 slices from 4-5 mice per group.*
